# Supplementary material for: Identification of A Novel Class of Benzofuran Oxoacetic Acid-Derived Ligands that Selectively Activate Cellular EPAC1
Source: Cells. 2019 Nov 12;8(11):1425. doi: 10.3390/cells8111425 (PMC6912754; doi:10.3390/cells8111425)
Supplement: Supplementary file 1 [file cells-08-01425-s001.zip › Supplementary Figures_SAR.pdf]

Supplementary Figure 1

R^1 oxo-acetic acid SAR

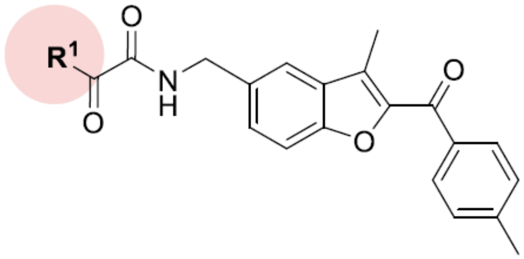

ESC Code	R^1	EPAC1 (pIC_{50})	EPAC1 (E_{max})	EPAC2 (pIC_{50})	EPAC2 (E_{max})
ESC1000851	**OH**	**4.9**	88	**5.1**	102
ESC1000852	**OEt**	< 4.0	16	< 4.0	18
ESC1001771	**OMe**	**4.3**	50	< 4.0	46
ESC1001772	**NHMe**	< 4.0	18	**4.0**	54
ESC1001793	**NMe_2**	< 4.0	-4	< 4.0	-2
ESC1001895	**CH_3**	< 4.0	8	< 4.0	1

Supplementary Figure 2

Alternative R^1 tethered acid SAR

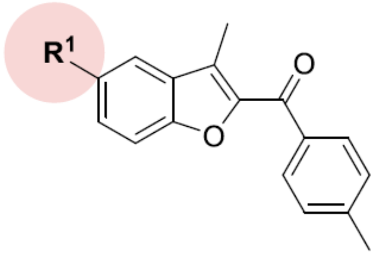

ESC Code	R^1	EPAC1 (pIC_{50})	EPAC1 (E_{max})	EPAC2 (pIC_{50})	EPAC2 (E_{max})
ESC1000853	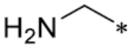	< 4.0	14	< 4.0	8
ESC1000854	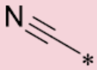	< 4.0	7	< 4.0	16
ESC1000915	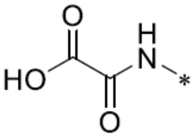	< 4.0	9	< 4.0	35
ESC1000960	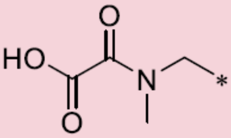	< 4.0	-6	< 4.0	29
ESC1001000	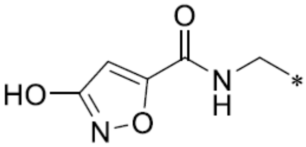	< 4.0	4	< 4.0	4
ESC1001622	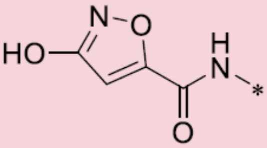	< 4.0	12	< 4.0	7
ESC1001634	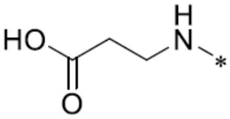	< 4.0	10	< 4.0	6
ESC1001650	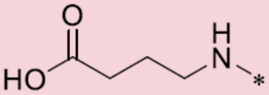	< 4.0	8	< 4.0	4
ESC1001696	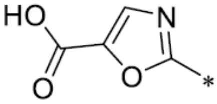	< 4.0	6	< 4.0	12
ESC1001702	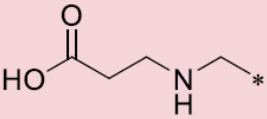	< 4.0	0	< 4.0	2
ESC1001792	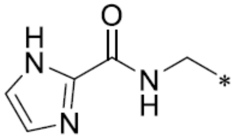	< 4.0	3	< 4.0	0
ESC1001889	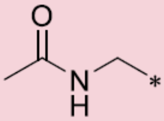	< 4.0	-2	< 4.0	-1
ESC1001918	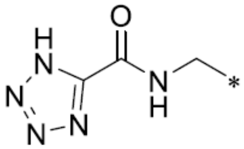	< 4.0	37	< 4.0	19

Supplementary Figure 3

Analogues with substitution at R^4 and R^5

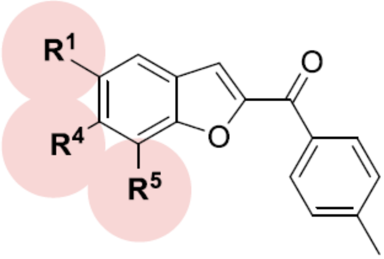

ESC Code	R^1	R^4	R^5	EPAC1 (pIC_{50})	EPAC2 (pIC_{50})
ESC1001742	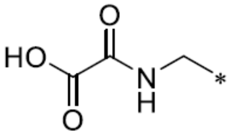	H	H	**5.0**	4.5
ESC1001896	H	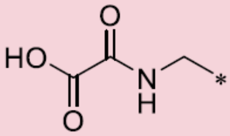	H	< 4.0	4.4
ESC1001954	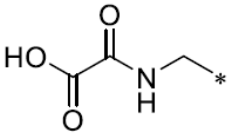	OMe	H	< 4.0	4.2
ESC1001955	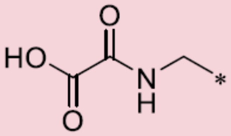	H	OMe	**4.9**	4.7
ESC1001956	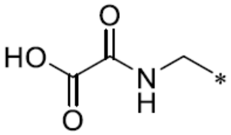	H	NH_2	4.3	4.5

Alternative heterocyclic core motifs

Supplementary Figure 4

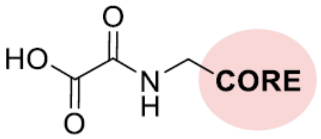

R^7

ESC Code	CORE	EPAC1 (pIC_{50})	EPAC2 (pIC_{50})
ESC1001264	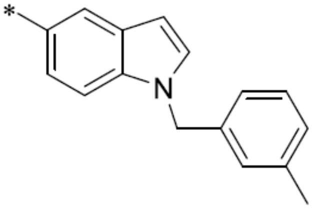	<4.0	**4.4**
ESC1001466	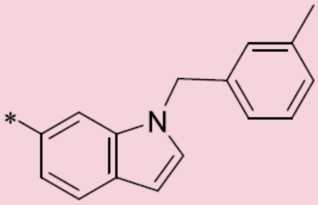	<4.0	**4.1**
ESC1001486	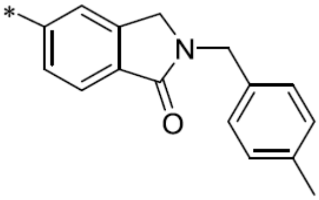	<4.0	<4.0
ESC1001487	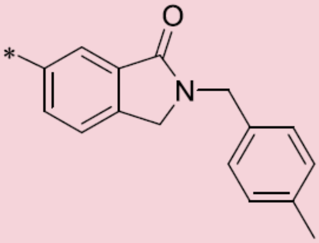	<4.0	<4.0
ESC1001549	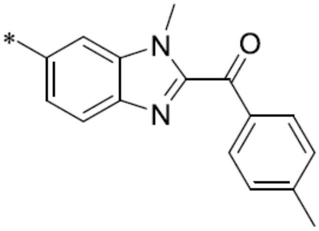	<4.0	<4.0
ESC1001568	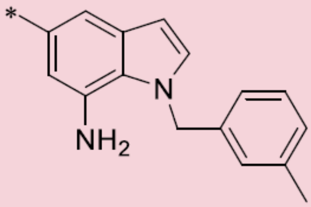	<4.0	<4.0
ESC1001596	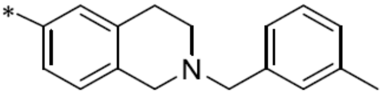	<4.0	<4.0
ESC1001597	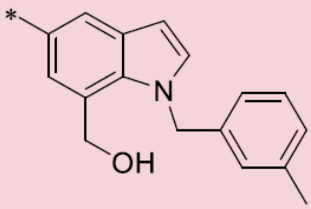	<4.0	<4.0

Supplementary Figure 5

Alternative R^2 linkers

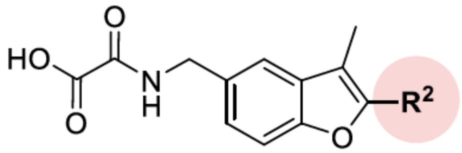

ESC Code	R^2	EPAC1 (pIC_{50})	EPAC2 (pIC_{50})	ESC Code	R^2	EPAC1 (pIC_{50})	EPAC2 (pIC_{50})
ESC1000851	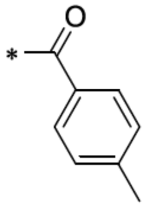	**4.9**	**5.1**	ESC1001581	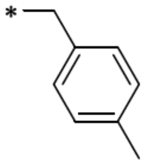	**4.0**	**4.6**
ESC1001536	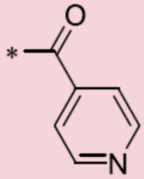	**4.3**	**4.7**	ESC1001547	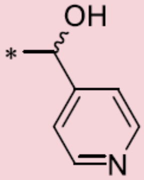	< 4.0	**4.7**

Supplementary Figure 6

R^2 aryl and heteroaryl ketone substituents

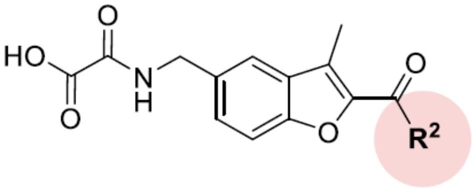

ESC Code	R^2	EPAC1 (pIC_{50})	EPAC2 (pIC_{50})	ESC Code	R^2	EPAC1 (pIC_{50})	EPAC2 (pIC_{50})
ESC1000851	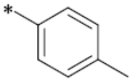	4.9	5.1	ESC1001548	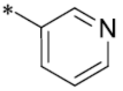	4.3	4.7
ESC1000906	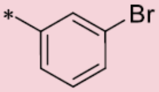	4.6	5.1	ESC1001595	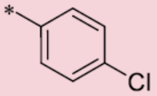	4.9	5.2
ESC1000907	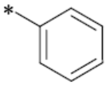	4.7	4.8	ESC1001602	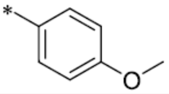	4.9	4.9
ESC1001536	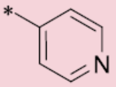	4.3	4.7	ESC1001604	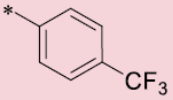	4.8	5.1
ESC1001621	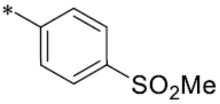	4.8	4.7				

R^2 cycloalkyl and heteroalkyl ketone substituents

ESC Code	R^3	R^2	EPAC1 (pIC_{50})	EPAC2 (pIC_{50})	Fsp^3	LE	LipE
ESC1000851	Me	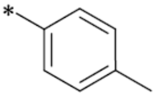	4.9	5.1	0.15	0.26	2.6
ESC1001531	Me	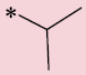	4.1	4.4	0.31	0.26	2.8
ESC1001653	Me	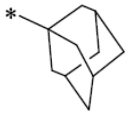	4.8	**5.8**	**0.52**	0.23	2.2
ESC1001866	i-Pr	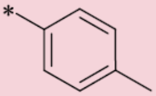	**5.4**	4.5	0.23	0.27	2.5
ESC1001908	i-Pr	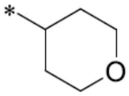	4.7	4.1	**0.45**	0.24	3.6
ESC1001920	i-Pr	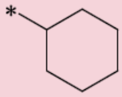	5.0	4.7	**0.48**	0.26	2.1

Supplementary Figure 7

R^2 Amide substituents

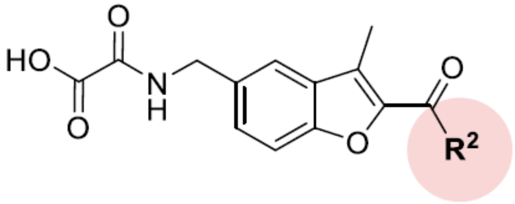

ESC Code	R^2	EPAC1 (pIC_{50})	EPAC2 (pIC_{50})
ESC1001605	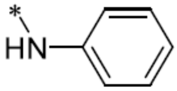	4.4	4.6
ESC1001607	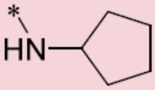	4.1	4.1
ESC1001608	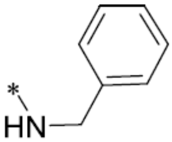	<4.0	<4.0
ESC1001609	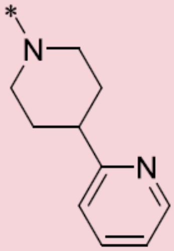	<4.0	<4.0
ESC1001610	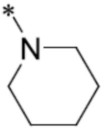	<4.0	<4.0
ESC1001693	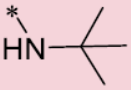	<4.0	4.4
ESC1001694	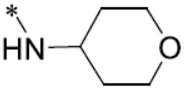	<4.0	4.3
ESC1001711	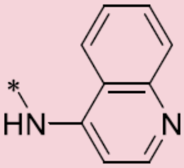	4.7	5.1
ESC1001876	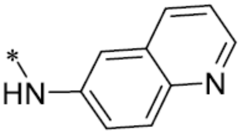	4.6	5.1
ESC1001877	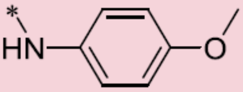	4.4	4.6
ESC1001878	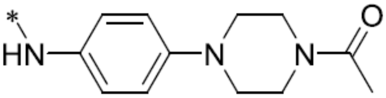	4.4	4.7

Supplementary Figure 8

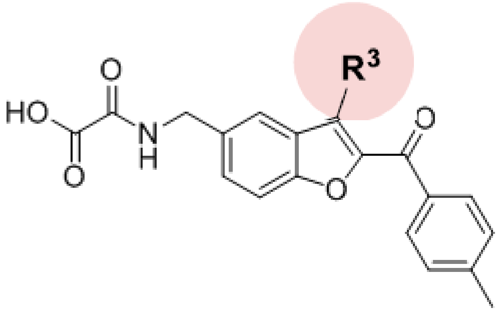

3-Substituted benzofuran analogues

	ESC Code	R^3	EPAC1 (pIC_{50})	EPAC2 (pIC_{50})
SY000 →	ESC1000851	Me	**4.9**	5.1
	ESC1001588	O*i*-Pr	**4.2**	4.4
	ESC1001628	OMe	**4.5**	4.6
	ESC1001652	Et	**5.3**	5.3
	ESC1001742	H	**5.0**	4.5
	ESC1001860	*c*-Pr	**5.0**	4.8
SY009 →	ESC1001866	*i*-Pr	**5.4**	4.5
	ESC1001867	*i*-Bu	**5.2**	4.7
	ESC1001903	*c*-Hex	**5.0**	4.3
	ESC1001919	4Cl-Ph	**4.9**	4.1
