# Supplementary material for: Identification of A Novel Class of Benzofuran Oxoacetic Acid-Derived Ligands that Selectively Activate Cellular EPAC1
Source: Cells. 2019 Nov 12;8(11):1425. doi: 10.3390/cells8111425 (PMC6912754; doi:10.3390/cells8111425)
Supplement: Supplementary file 1 [file cells-08-01425-s001.zip › SY009 synthesis and spectroscopic data.pdf]

SY009 Synthesis and Data

1-(5-Bromo-2-hydroxyphenyl)-2-methylpropan-1-one 2

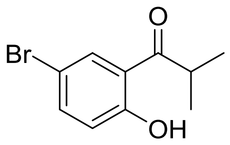

2

iso-Propylmagnesium bromide (76 mL of a 2.9 M solution in 2-MeTHF, 22.5 mmol) was added dropwise to a stirred solution of 5-bromo-2-hydroxybenzonitrile (1.49 g, 7.5 mmol) in THF (10 mL) at 0 °C. The resulting solution was allowed to warm to rt and stirred for a further 2 h. The solution was then cooled to 0 °C, and water (1.5 mL) and 6 M HCl (2 mL) were added. Then, the mixture was stirred and heated at reflux for 1 h before being allowed to cool to rt. EtOAc (40 mL) and water (10 mL) were added and the layers separated. The organic layer was washed with water (10 mL) and brine (20 mL), dried ($MgSO_4$), and evaporated under reduced pressure to give the crude product. Purification by flash column chromatography on silica with petroleum ether:EtOAc (90:10) as eluent gave ketone **2** as a yellow oil (1.66 g, 91%); R_F 0.6 (petroleum ether:EtOAc 90:10); IR (film) 2973, 2935, 2873, 1639 (C=O str), 1606, 1568, 1466, 1405, 1385, 1361, 1338, 1287, 1266, 1236, 1191, 1159, 1100, 1080, 984, 875, 825 cm^{-1}; ^{1}H NMR (300 MHz, $CDCl_3$) δ 12.45 (s, 1H, OH), 7.92 (d, *J* = 2.5 Hz, 1H, Ar), 7.57 (dd, *J* = 9.0 , 2.5 Hz, 1H, Ar), 6.94 (d, *J* = 9.0 Hz, 1H, Ar), 3.57 (septet, *J* = 7.0 Hz, 1H, CH), 1.29 (d, *J* = 7.0 Hz, 6H, CH*Me*$_2$); ^{13}C NMR (75.5 MHz, $CDCl_3$) δ 209.9 (C), 162.1 (C), 138.9 (CH), 132.1 (CH), 120.8 (CH), 119.4 (C), 110.4 (C), 35.1 (CH), 19.2 (CH_3). Spectroscopic data consistent with those reported in the literature (G. Bononi, C. Granchi, M. Lapillo, M. Giannotti, D. Nieri, S. Fortunato, M. El Boustani, I. Caligiuri, G. Poli, K. E. Carlson, S. H. Kim, M. Macchia, A. Martinelli, F. Rizzolio, A. Chicca, J. A. Katzenellenbogen, F. Minutolo and T. Tuccinardi, *Eur. J. Med. Chem.*, 2018, **157**, 817–836).

(5-Bromo-3-*iso*-propylbenzofuran-2-yl)(*p*-tolyl)methanone 4

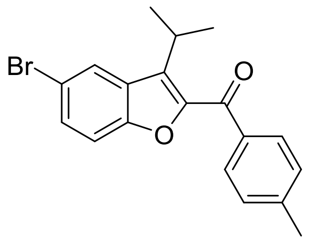

4

A solution of 1-(5-bromo-2-hydroxyphenyl)-2-methylpropan-1-one **2** (2.78 g, 11.4 mmol), 2-bromo-1-(*p*-tolyl)ethenone (2.92 g, 13.7 mmol) and K_2CO_3 (3.16 g, 22.9 mmol) in DMF (20 mL) was stirred and heated at 60 °C for 2 h. The resulting solution was allowed to cool to rt and EtOAc (40 mL) and water

(30 mL) were added. The layers were separated, and the organic layer was washed with water (30 mL), brine (30 mL), dried ($MgSO_4$) and evaporated under reduced pressure. PhMe (30 mL) and *p*-TsOH (209 mg, 1.1 mmol) were added and the resulting soluton was stirred and heated at reflux under a Dean-Starck trap for 16 h. The resulting solution was allowed to cool to rt and CH_2Cl_2 (30 mL) was added. The layers were separated, and the organic layer was washed with saturated $NaHCO_{3(aq)}$ (2 × 30 mL), brine (30 mL), dried ($MgSO_4$) and evaporated under reduced pressure to give the crude product. Purification by flash column chromatography on silica with petroleum ether:EtOAc (99:1) as eluent gave product **4** as an off white solid (3.92 g, 96%); R_F 0.2 (petroleum ether:EtOAc 99:1); mp 110-113 °C; IR (solid) 2966, 2929, 2870, 1641 (C=O str), 1601, 1562, 1464, 1446, 1356, 1314, 1305, 1263, 1232, 1211, 1185, 1173, 1156, 1129, 1109, 1061, 1045, 978, 965, 884, 874, 861, 847, 837, 807 cm^{-1}; ^1H NMR (400 MHz, $CDCl_3$) δ 8.03 (dd, *J* = 2.0, 0.5 Hz, 1H, Ar), 7.94 (d, *J* = 8.0 Hz, 2H, Ar), 7.50 (dd, *J* = 9.0, 2.0 Hz, 1H, Ar), 7.39 (dd, *J* = 9.0, 0.5 Hz, 1H, Ar), 7.30 (d, *J* = 8.0 Hz, 2H, Ar), 3.97 (septet, *J* = 7.0 Hz, 1H, CH), 2.43 (s, 3H, Me), 1.47 (d, *J* = 7.0 Hz, 6H, CH*Me*$_2$); ^{13}C NMR (75.5 MHz, $CDCl_3$) δ 185.7 (C), 153.1 (C), 147.9 (C), 143.7 (C), 135.1 (C), 134.6 (C), 130.3 (CH), 130.0 (CH), 129.0 (C), 129.0 (CH), 125.6 (CH), 116.0 (C), 113.9 (CH), 25.3 (CH), 22.0 (CH_3), 21.7 (CH_3); LRMS (TOF MS ASAP+) *m/z* 357 ($[M + H]^+$), 279 [$(M – Br)^+$]; HRMS (TOF MS ASAP+) *m/z* $C_{19}H_{18}O_2Br$ ($[M + H]^+$) calcd for 357.0490, found 357.0488.

3-*iso*-Propyl-2-(4-methylbenzoyl)benzofuran-5-carbonitrile 5

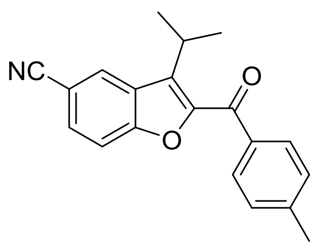

5

A solution of **4** (2.10 g, 5.88 mmol), $Zn(CN)_2$ (829 mg, 7.06 mmol), Pd_2dba_3 (375 mg, 0.41 mmol) and dppf (456 mg, 0.82 mmol) in DMF (30 mL) was stirred at heated at 120 °C under a nitrogen atmosphere supplied *via* a Shlenck line fitted with a $CuSO_4$ bubbler for 4 h. The resulting solution was allowed to cool to rt and stirred for a further 60 h. Then, the mixture was filtered over Celite®, washing with EtOAc. EtOAc (50 mL) was added to the filtrate and the resulting organic solution was washed with dilute $NH_4OH_{(aq)}$ (2 × 40 mL), brine (40 mL), dried ($MgSO_4$) and evaporated under reduced pressure to give the crude product. Purification by flash column chromatography on silica with petroleum ether:EtOAc (95:5) as eluent gave product **5** as an off-white solid (1.59 g, 89%); R_F 0.3 (petroleum

ether:EtOAc 95:5); mp 138-140 °C; IR (solid) 2923, 2223 (C≡N), 1645 (C=O), 1607, 1557, 1456, 1404, 1362, 1313, 1272, 1239, 1183, 1151, 1095, 1053, 977, 904, 880 cm^{-1}; ^1H NMR (300 MHz, $CDCl_3$) δ 8.25 (d, *J* = 1.5 Hz, 1H, Ar), 7.92 (d, *J* = 8.0 Hz, 2H, Ar), 7.70 (dd, *J* = 8.5, 1.5 Hz, 1H, Ar), 7.62 (d, *J* = 8.5 Hz, 1H, Ar), 7.33 (d, *J* = 8.0 Hz, 2H, Ar), 3.94 (sept, *J* = 7.0 Hz, 1H, CH), 2.46 (s, 3H, Me), 1.47 (d, *J* = 7.0 Hz, 6H, CHMe_2); ^{13}C NMR (75.5 MHz, $CDCl_3$) δ 185.7 (C), 156.0 (C), 148.7 (C), 144.4 (C), 134.9 (C), 134.7 (C), 130.4 (CH), 130.1 (CH), 129.3 (CH), 128.6 (CH), 127.9 (C), 119.2 (C), 113.9 (CH), 107.2 (C), 25.4 (CH), 22.2 (CH_3), 21.9 (CH_3); LRMS (TOF MS ASAP+) *m/z* 304 ($[M + H]^+$), 279 ($[M - CN]^+$); HRMS (TOF MS ASAP+) *m/z* $C_{20}H_{18}NO_2$ ($[M + H]^+$) calcd for 304.1338, found 304.1339.

(5-(Aminomethyl)-3-*iso*-propylbenzofuran-2-yl)(*p*-tolyl)methanol 6

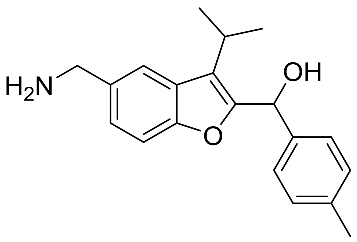

6

3-*iso*-propyl-2-(4-methylbenzoyl)benzofuran-5-carbonitrile **5** (100 mg, 0.33 mmol) in THF (5 mL) was added dropwise to a stirred suspension of $LiAlH_4$ (76 mg, 1.98 mmol) in THF (5 mL) at 0 °C. The resulting solution was stirred and heated at reflux for 16 h. The solution was then cooled to 0 °C, and quenched sequentially with water (0.08 mL), 1 M $NaOH_{(aq)}$ (0.16 mL), and water (0.08 mL), dried (Na_2SO_4), filtered through Celite® and washed through with CH_2Cl_2:MeOH (95:5), and evaporated under reduced pressure to give the crude product. Purification by flash column chromatography on silica with CH_2Cl_2→ CH_2Cl_2:MeOH (98:2) → CH_2Cl_2:MeOH:$NH_4OH_{(aq)}$ (97:2:1) as eluent gave product **6** as a colourless oil (50 mg, 49%); R_F 0.3 (CH_2Cl_2:MeOH:$NH_4OH_{(aq)}$ 97:2:1); IR (film) 3359 (N-H str), 3298 (N-H str), 3022 (OH), 2962, 2926, 2869, 1588, 1511, 1471, 1445, 1383, 1364, 1327, 1255, 1174, 1107, 1091, 1049, 1030, 1019, 909, 890, 873, 817 cm^{-1}; ^1H NMR (300 MHz, $CDCl_3$) δ 7.54 (d, *J* = 1.0 Hz, 1H, Ar), 7.33 (d, *J* = 8.0 Hz, 2H, Ar), 7.30 (d, *J* = 8.5 Hz, 1H, Ar), 7.14 (d, *J* = 8.0 Hz, 2H, Ar), 7.10 (dd, *J* = 8.5, 1.0 Hz, 1H, Ar), 6.01 (s, 1H, CHOH), 3.86 (s, 2H, CH_2), 3.27 (septet, *J* = 7.0 Hz, 1H, CH), 2.63 (br s, 2H, NH_2), 2.33 (s, 3H, CMe), 1.42 (d, *J* = 7.0 Hz, 3H, CH$Me_A$$Me_B$), 1.40 (d, *J* = 7.0 Hz, 3H, CH$Me_A$$Me_B$); ^{13}C NMR (101 MHz, $CDCl_3$) δ 153.8 (C), 151.9 (C), 138.7 (C), 137.4 (C), 136.8 (C), 129.2 (CH), 128.0 (C), 126.3 (CH), 123.6 (CH), 122.1 (C), 119.6 (CH), 111.7 (CH), 68.0 (CH), 46.6 (CH_2), 25.3 (CH), 22.7 (CH_3), 22.6 (CH_3), 21.2 (CH_3).

(5-(Aminomethyl)-3-*iso*-propylbenzofuran-2-yl)(*p*-tolyl)methanone 7

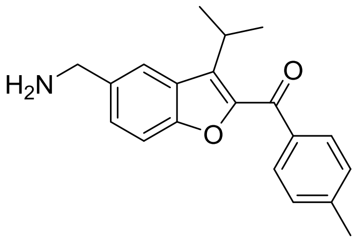

7

A solution of (5-(aminomethyl)-3-*iso*-propylbenzofuran-2-yl)(*p*-tolyl)methanol **6** (203 mg, 0.66 mmol), MnO_2 (856 mg, 9.84 mmol) in CH_2Cl_2 (5 mL) was stirred at rt for 7 h and the reaction monitored by TLC. The solution was filtered through Celite® and washed through with CH_2Cl_2:MeOH (95:5) and evaporated under reduced pressure to give the crude product. Purification by flash column chromatography on silica with CH_2Cl_2→ CH_2Cl_2:MeOH (98:2) → CH_2Cl_2:MeOH:$NH_4OH_{(aq)}$ (96:3:1) as eluent gave product **7** as an yellow oil (43 mg, 21%); R_F 0.2 (CH_2Cl_2:MeOH $NH_4OH_{(aq)}$ 96:3:1); IR (film) 2966, 2929, 2360, 1698, 1644 (C=O), 1606, 1557, 1462, 1363, 1317, 1266, 1235, 1183, 1093, 1053, 978, 911, 876, 814 cm^{-1}; ^1H NMR (300 MHz, $CDCl_3$) δ 7.94 (d, *J* = 8.0 Hz, 2H, Ar), 7.81 (d, *J* = 1.5 Hz, 1H, Ar), 7.49 (d, *J* = 8.5 Hz, 1H, Ar), 7.42 (dd, *J* = 8.5, 1.5 Hz, 1H, Ar), 7.31 (d, *J* = 8.0 Hz, 2H, Ar), 4.00 (s, 2H, CH_2), 3.99 (sept, *J* = 7.0 Hz, 1H, CH), 2.45 (s, 3H, CMe), 1.82 (br s, 2H, NH_2), 1.49 (d, *J* = 7.0 Hz, 6H, CHMe_2); ^{13}C NMR (75.5 MHz, $CDCl_3$) δ 186.3 (C), 153.9 (C), 147.6 (C), 143.7 (C), 138.1 (C), 135.8 (C), 135.6 (C), 130.1 (CH), 129.1 (CH), 127.5 (C), 127.4 (CH), 121.4 (CH), 112.7 (CH), 46.7 (CH_2), 25.6 (CH), 22.3 (CH_3), 21.9 (CH_3).

Ethyl 2-(((3-*iso*-propyl-2-(4-methylbenzoyl)benzofuran-5-yl)methyl)amino)-2-oxoacetate 8

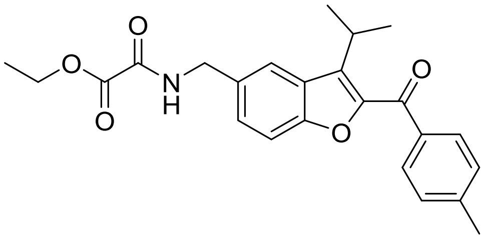

8

A solution of **7** (43 mg, 0.14 mmol) and Et_3N (39 µL, 0.28 mmol) in CH_2Cl_2 (4 mL) was stirred at 0 °C and ethyl oxalylchloride (18.7 µL, 0.17 mmol) was added dropwise. The resulting solution was stirred at 0

°C for 10 min then allowed to warm to rt and stirred for a further 2 h. Then, CH_2Cl_2 (5 mL) was added and the resulting solution was washed with water (5 mL), dried ($MgSO_4$) and evaporated under reduced pressure to give the crude product. Purification by flash column chromatography on silica with petroleum ether:EtOAc (80:20) as eluent gave product **8** as a colourless oil (51 mg, 89%); R_F 0.2 (petroleum ether:EtOAc 80:20); IR (film) 3316 (N-H), 2967, 2927, 2872, 1734 (C=O), 1690 (C=O), 1642 (C=O), 1607, 1555, 1463, 1367, 1299, 1266, 1211, 1095, 1055, 1020, 974, 911, 876, 832 cm^{-1}; ^1H NMR (300 MHz, $CDCl_3$) δ 7.92 (d, *J* = 8.0 Hz, 2H, Ar), 7.81 (d, *J* = 1.5 Hz, 1H, Ar), 7.52 (br s, 1H, NH), 7.50 (d, *J* = 8.5 Hz, 1H, Ar), 7.40 (dd, *J* = 8.5, 1.5 Hz, 1H, Ar), 7.31 (d, *J* = 8.0 Hz, 2H, Ar), 4.63 (d, *J* = 6.0 Hz, 2H, CH_2NH), 4.35 (q, *J* = 7.0 Hz, 2H, OCH_2CH_3), 3.96 (septet, *J* = 7.0 Hz 1H, CH), 2.45 (s, 3H, CMe), 1.47 (d, *J* = 7.0 Hz, 6H, CH*Me*$_2$), 1.38 (t, *J* = 7.0 Hz, 3H, OCH_2CH_3); ^{13}C NMR (75.5 MHz, $CDCl_3$) δ 186.2 (C), 160.7 (C), 156.6 (C), 154.2 (C), 147.8 (C), 143.8 (C), 135.5 (C), 135.4 (C), 131.8 (C), 130.1 (CH), 129.1 (CH), 127.9 (CH), 127.6 (C), 123.0 (CH), 113.1 (CH), 63.5 (CH_2), 44.2 (CH_2), 25.5 (CH), 22.2 (CH_3), 21.9 (CH_3), 14.1 (CH_3).

2-(((3-*iso*-Propyl-2-(4-methylbenzoyl)benzofuran-5-yl)methyl)amino)-2-oxoacetic acid 9 (SY009)

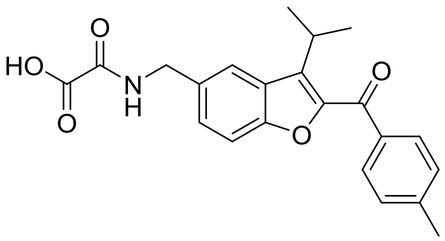

9

NaOH (8 mg, 0.21 mmol) was added to a stirred solution of **8** (43 mg, 0.11 mmol) in 1:1 MeOH:THF (4 mL) at rt, and the resulting solution was stirred at rt for 20 min, monitoring the reaction by TLC. Then, 1M $HCl_{(aq)}$ (0.5 mL) and water (10 mL) were added. The organic solvents were removed under reduced pressure and the remaining aqueous solution was extracted with EtOAc (3 × 10 mL). The combined organic layers were dried ($MgSO_4$) and evaporated under reduced pressure to give product **9** as an off-white solid (34 mg, 85%); mp 138-141 °C; IR (solid) 3283 (NH), 2966, 2871, 1760 (C=O), 1686 (C=O), 1636 (C=O), 1607, 1549, 1464, 1409, 1355, 1312, 1286, 1268, 1237, 1186, 1174, 1094, 1054, 1009, 973, 908, 875, 834, 810 cm^{-1}; ^1H NMR (300 MHz, Acetone-d_6) δ 8.90 (br s, 1H, NH), 8.04 (s, 1H, Ar), 7.93 (d, *J* = 8.0 Hz, 2H, Ar), 7.56 (m, 2H, Ar), 7.38 (d, *J* = 8.0 Hz, 2H, Ar), 4.66 (d, *J* = 6.5 Hz, 2H, CH_2N), 3.96 (septet, *J* = 7.0 Hz, 1H, CH), 2.44 (s, 3H, CMe), 1.47 (d, *J* = 7.0 Hz, 6H, CH*Me*$_2$); ^{13}C NMR (75.5 MHz, Acetone-d_6) δ 186.3 (C), 166.9 (C), 161.8 (C), 154.7 (C), 148.3 (C), 144.5 (C), 136.3 (C), 135.7 (C), 134.4

(C), 130.7 (CH), 129.8 (CH), 128.8 (CH), 127.9 (C), 123.4 (CH), 113.2 (CH), 44.2 (CH_2), 26.1 (CH), 22.2 (CH_3), 21.6 (CH_3).

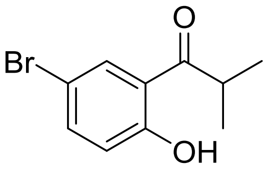

O
Br
OH

2

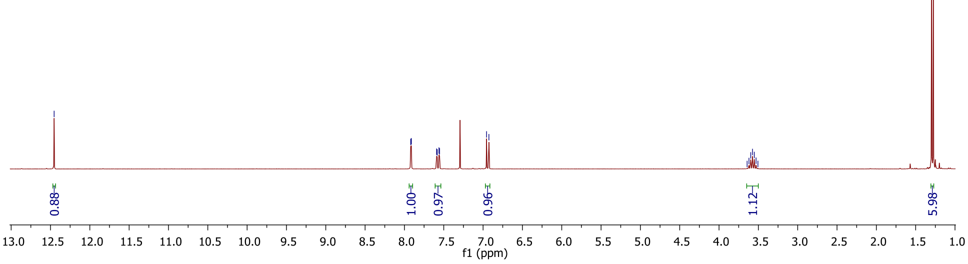

12.45
7.92
7.91
7.59
7.58
7.56
7.55
6.96
6.93
3.64
3.62
3.60
3.57
3.55
3.53
3.51
1.30
1.28
0.88
1.00
0.97
0.96
1.12
5.98
13.0 12.5 12.0 11.5 11.0 10.5 10.0 9.5 9.0 8.5 8.0 7.5 7.0 6.5 6.0 5.5 5.0 4.5 4.0 3.5 3.0 2.5 2.0 1.5 1.0
f1 (ppm)

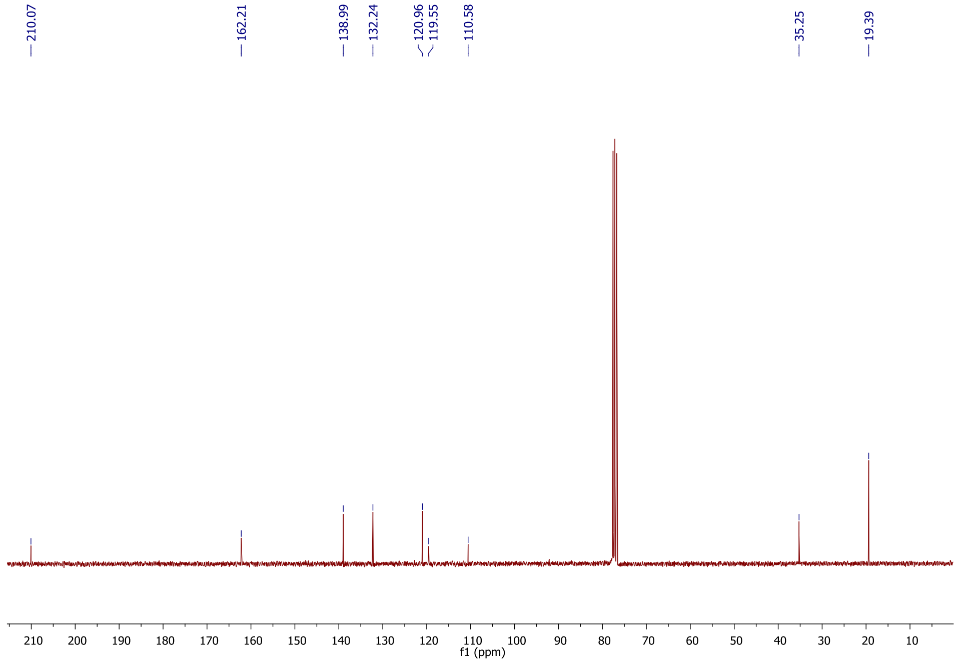

210.07
162.21
138.99
132.24
120.96
119.55
110.58
35.25
19.39
210 200 190 180 170 160 150 140 130 120 110 100 90 80 70 60 50 40 30 20 10
f1 (ppm)

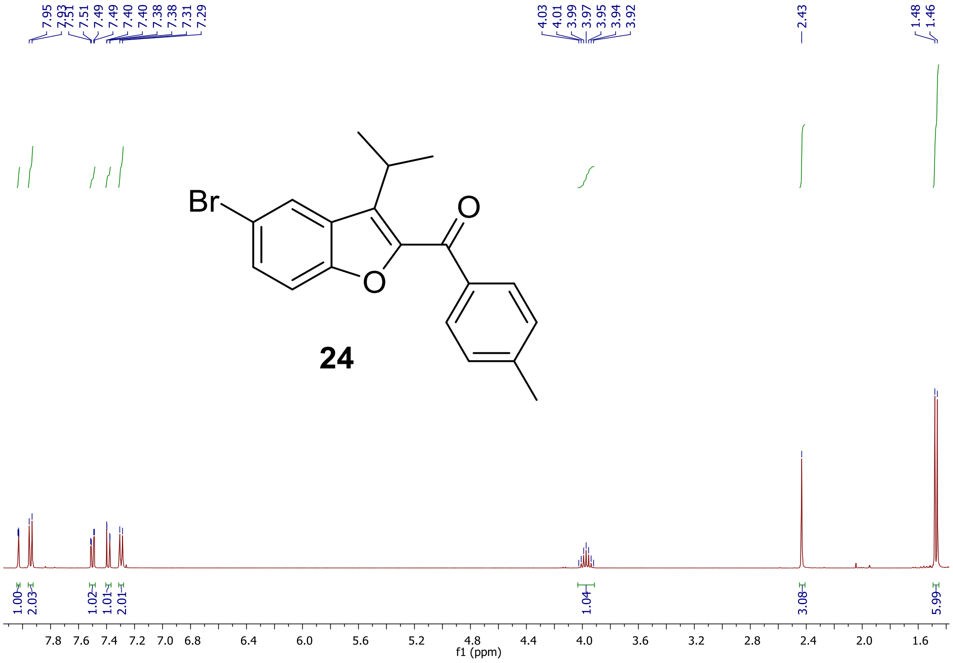

7.95
7.93
7.51
7.51
7.49
7.49
7.40
7.40
7.38
7.38
7.31
7.29
4.03
4.01
3.99
3.97
3.95
3.94
3.92
2.43
1.48
1.46
Br
O
O
24
1.00
2.03
1.02
1.01
2.01
1.04
3.08
5.99
7.8 7.6 7.4 7.2 7.0 6.8 6.4 6.0 5.6 5.2 4.8 4.4 4.0 3.6 3.2 2.8 2.4 2.0 1.6
f1 (ppm)

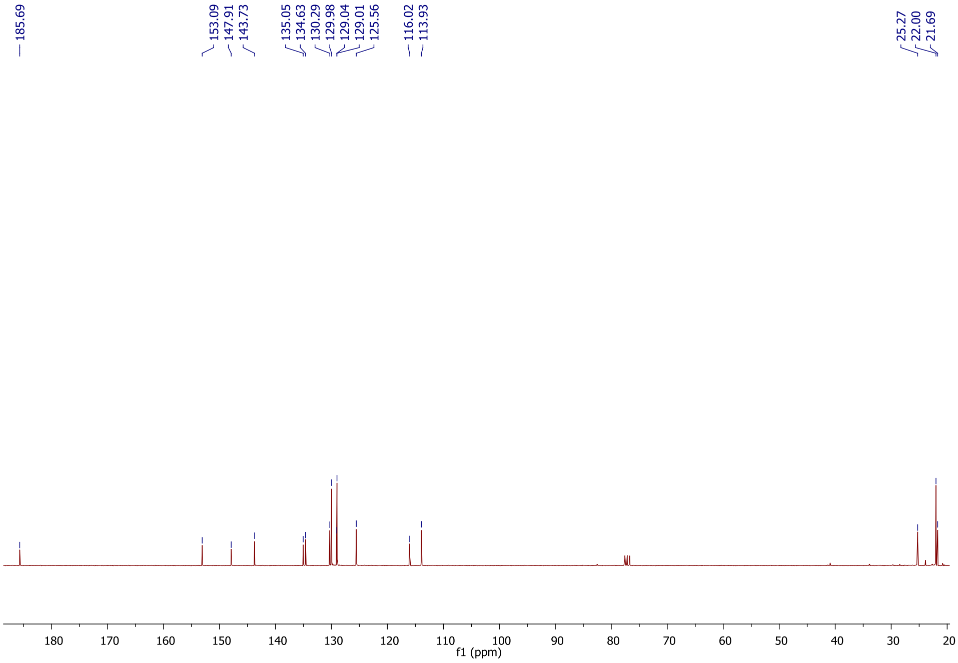

185.69
153.09
147.91
143.73
135.05
134.63
130.29
129.98
129.04
129.01
125.56
116.02
113.93
25.27
22.00
21.69
180 170 160 150 140 130 120 110 100 90 80 70 60 50 40 30 20
f1 (ppm)

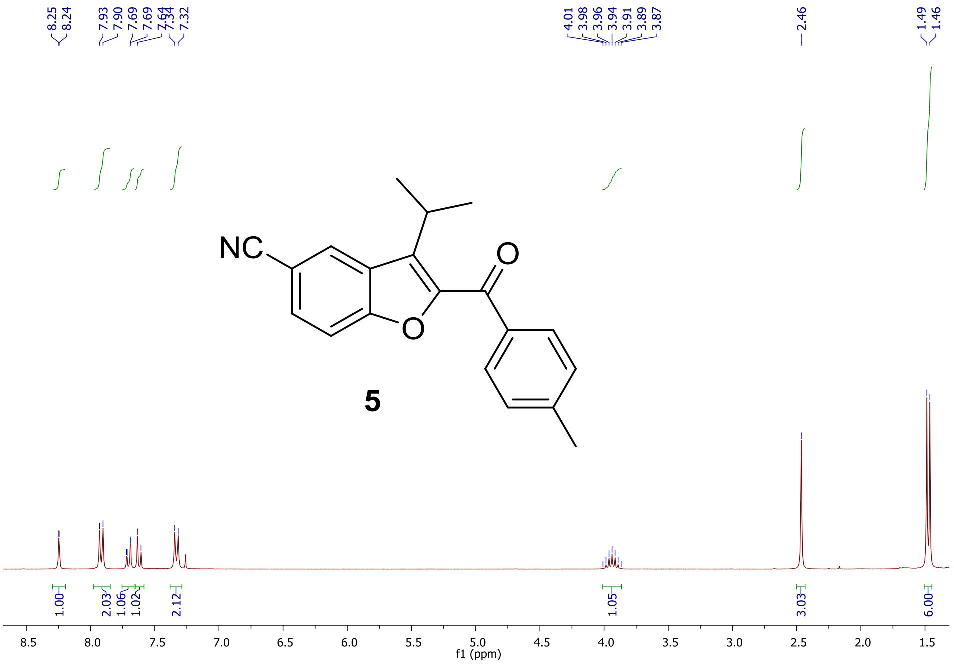
8.25
8.24
7.93
7.90
7.69
7.69
7.64
7.34
7.32
4.01
3.98
3.96
3.94
3.91
3.89
3.87
2.46
1.49
1.46
NC
O
O
5
1.00
2.03
1.06
1.02
2.12
1.05
3.03
6.00
8.5
8.0
7.5
7.0
6.5
6.0
5.5
5.0
4.5
4.0
3.5
3.0
2.5
2.0
1.5
f1 (ppm)

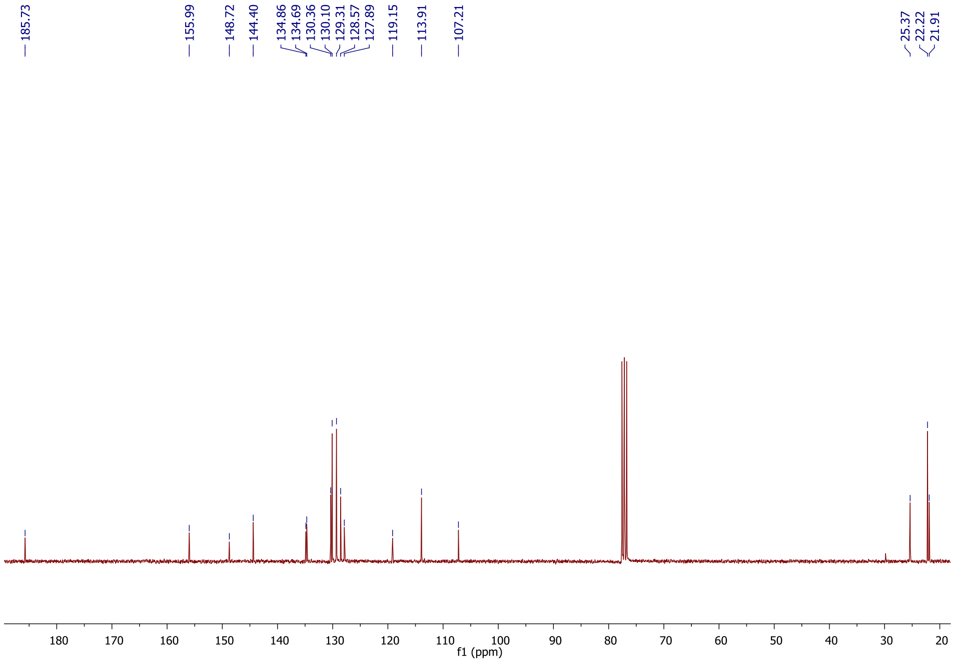
185.73
155.99
148.72
144.40
134.86
134.69
130.36
130.10
129.31
128.57
127.89
119.15
113.91
107.21
25.37
22.22
21.91
180
170
160
150
140
130
120
110
100
90
80
70
60
50
40
30
20
f1 (ppm)

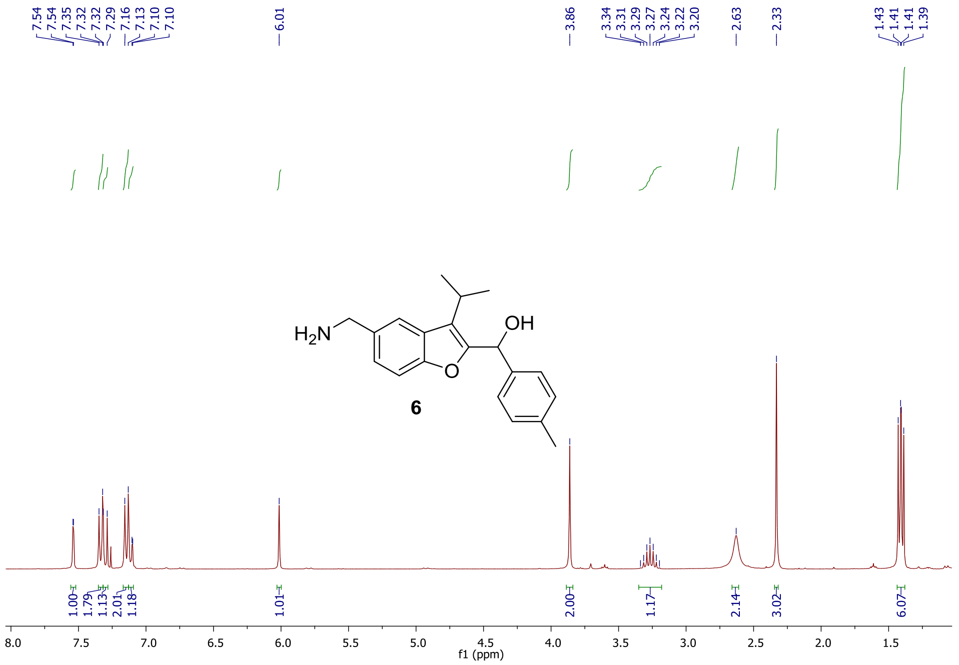
7.54
7.54
7.35
7.32
7.32
7.29
7.16
7.13
7.10
7.10
6.01
3.86
3.34
3.31
3.29
3.27
3.24
3.22
3.20
2.63
2.33
1.43
1.41
1.41
1.39
H_2N
OH
O
6
1.00
1.79
1.13
2.01
1.18
1.01
2.00
1.17
2.14
3.02
6.07
8.0 7.5 7.0 6.5 6.0 5.5 5.0 4.5 4.0 3.5 3.0 2.5 2.0 1.5
f1 (ppm)

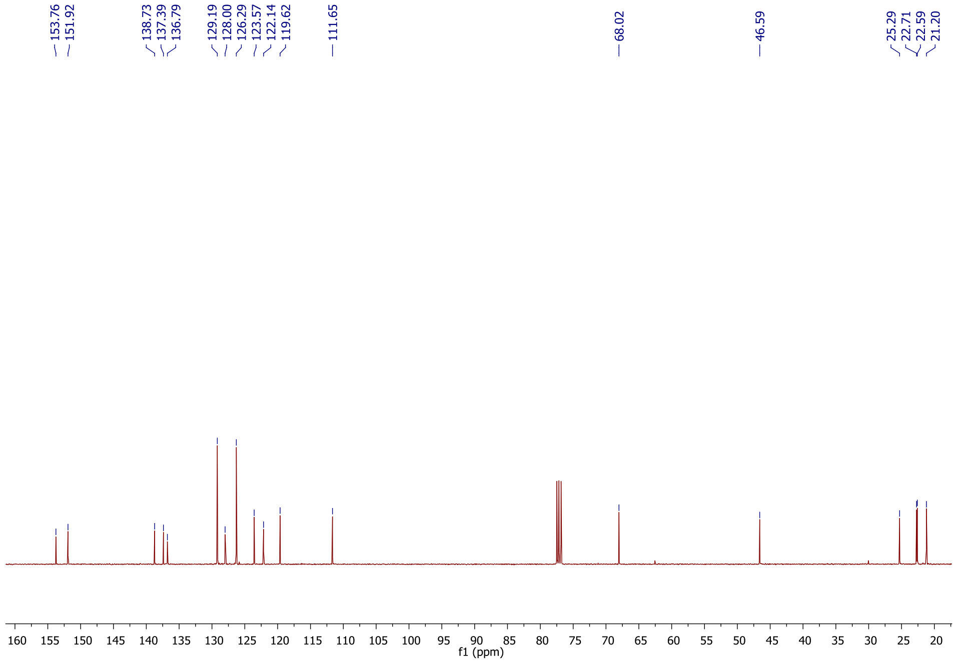
153.76
151.92
138.73
137.39
136.79
129.19
128.00
126.29
123.57
122.14
119.62
111.65
68.02
46.59
25.29
22.71
22.59
21.20
160 155 150 145 140 135 130 125 120 115 110 105 100 95 90 85 80 75 70 65 60 55 50 45 40 35 30 25 20
f1 (ppm)

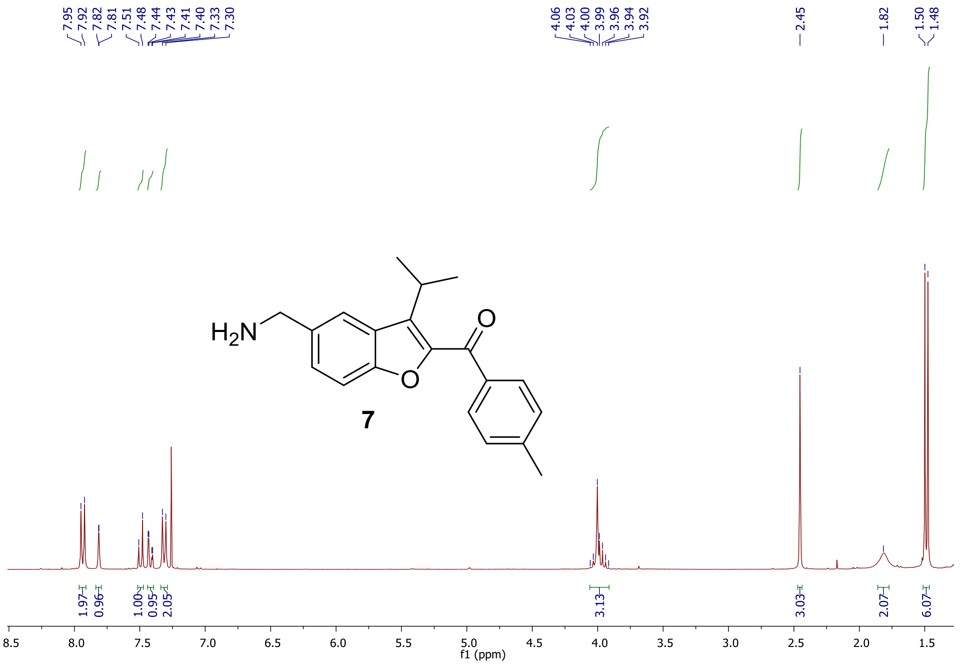

7.95
7.92
7.82
7.81
7.51
7.48
7.44
7.43
7.41
7.40
7.33
7.30
4.06
4.03
4.00
3.99
3.96
3.94
3.92
2.45
1.82
1.50
1.48
H2N
O
O
7
1.97
0.96
1.00
0.95
2.05
3.13
3.03
2.07
6.07
8.5
8.0
7.5
7.0
6.5
6.0
5.5
5.0
4.5
4.0
3.5
3.0
2.5
2.0
1.5
f1 (ppm)

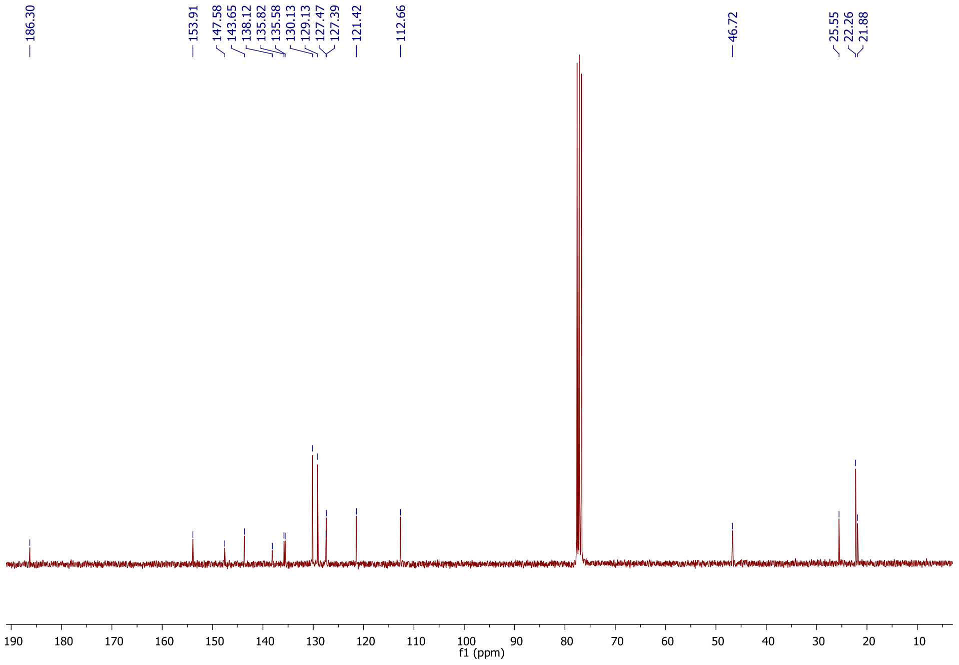

186.30
153.91
147.58
143.65
138.12
135.82
135.58
130.13
129.13
127.47
127.39
121.42
112.66
46.72
25.55
22.26
21.88
190
180
170
160
150
140
130
120
110
100
90
80
70
60
50
40
30
20
10
f1 (ppm)

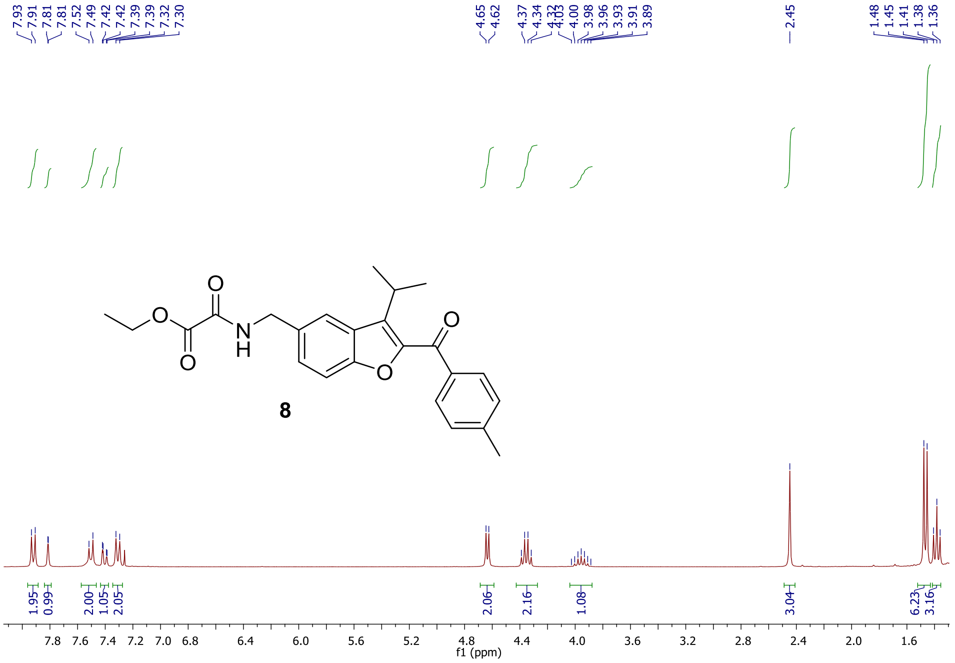
7.93 7.91 7.81 7.81 7.52 7.49 7.42 7.42 7.39 7.39 7.32 7.30
4.65 4.62 4.37 4.34 4.32 4.03 4.00 3.98 3.96 3.93 3.91 3.89
2.45
1.48 1.45 1.41 1.38 1.36
O
O
N
H
O
O
8
1.95 0.99 2.00 1.05 2.05 2.06 2.16 1.08 3.04 6.23 3.16
7.8 7.6 7.4 7.2 7.0 6.8 6.4 6.0 5.6 5.2 4.8 4.4 4.0 3.6 3.2 2.8 2.4 2.0 1.6
f1 (ppm)

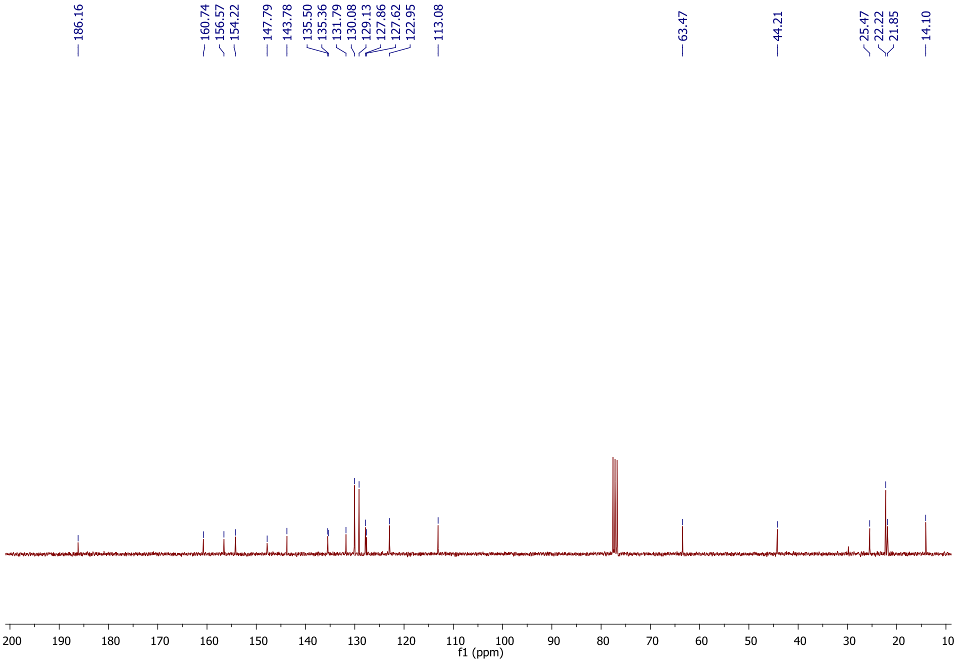
186.16
160.74 156.57 154.22 147.79 143.78 135.50 135.36 131.79 130.08 129.13 127.86 127.62 122.95 113.08
63.47
44.21
25.47 22.22 21.85 14.10
200 190 180 170 160 150 140 130 120 110 100 90 80 70 60 50 40 30 20 10
f1 (ppm)

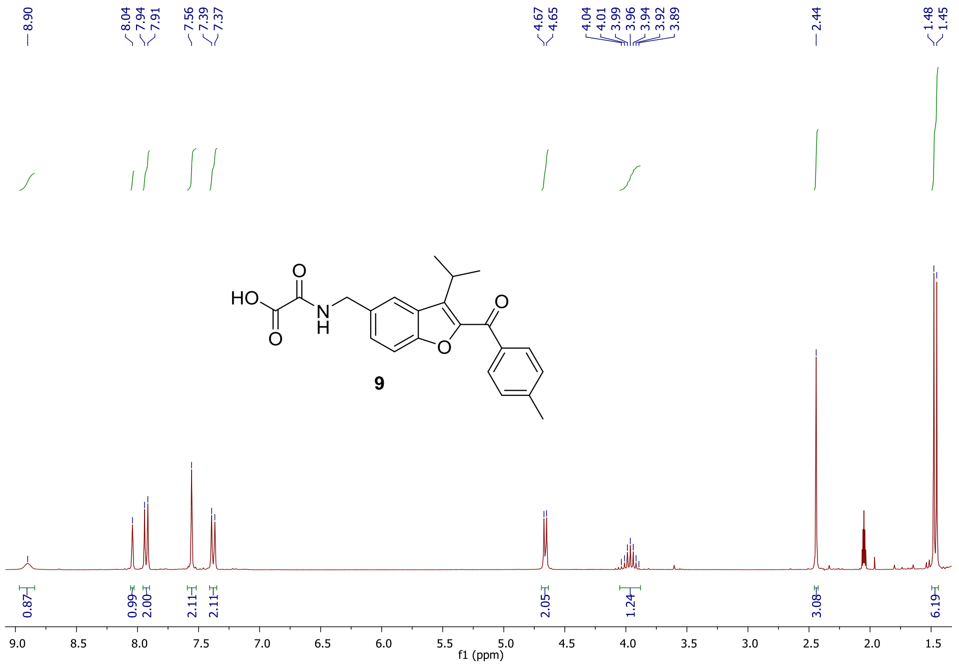

8.90
8.04
7.94
7.91
7.56
7.39
7.37
4.67
4.65
4.04
4.01
3.99
3.96
3.94
3.92
3.89
2.44
1.48
1.45
HO
O
N
H
O
O
9
0.87
0.99
2.00
2.11
2.11
2.05
1.24
3.08
6.19
9.0 8.5 8.0 7.5 7.0 6.5 6.0 5.5 5.0 4.5 4.0 3.5 3.0 2.5 2.0 1.5
f1 (ppm)

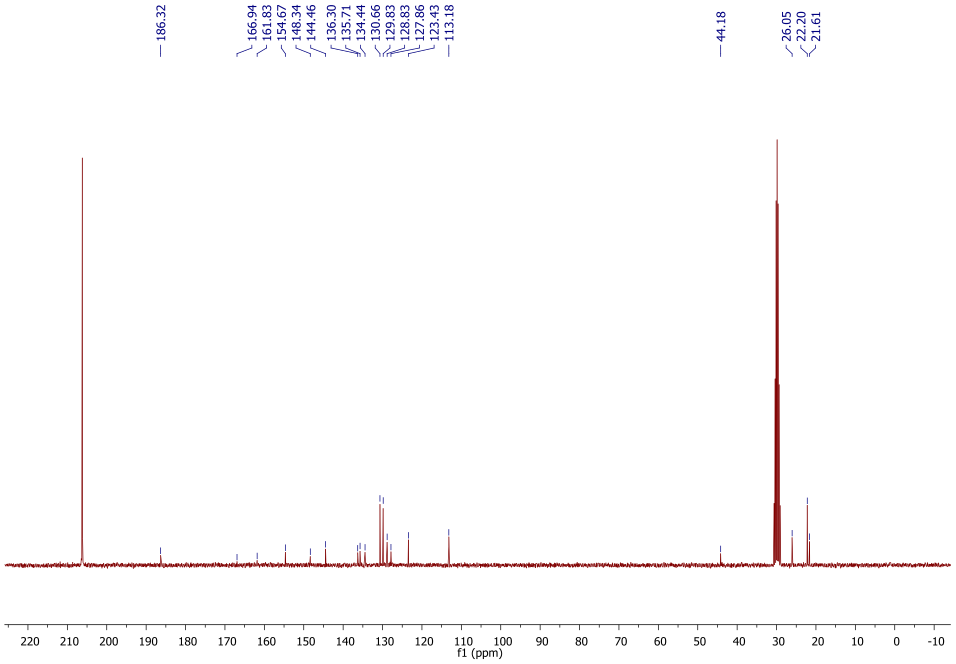

186.32
166.94
161.83
154.67
148.34
144.46
136.30
135.71
134.44
130.66
129.83
128.83
127.86
123.43
113.18
44.18
26.05
22.20
21.61
220 210 200 190 180 170 160 150 140 130 120 110 100 90 80 70 60 50 40 30 20 10 0 -10
f1 (ppm)
